# Aberrant populations of circulating T follicular helper cells and regulatory B cells underlying idiopathic pulmonary fibrosis

**DOI:** 10.1186/s12931-019-1216-6

**Published:** 2019-11-06

**Authors:** Yuichiro Asai, Hirofumi Chiba, Hirotaka Nishikiori, Ryuta Kamekura, Hayato Yabe, Shun Kondo, Satsuki Miyajima, Katsunori Shigehara, Shingo Ichimiya, Hiroki Takahashi

**Affiliations:** 1 Department of Respiratory Medicine and Allergology, Sapporo Medical University School of Medicine, 1-37, South 1-West 16, Chuo-ku, Sapporo, Hokkaido 060-8543 Japan; 2 Department of Human Immunology, Research Institute for Frontier Medicine, Sapporo Medical University School of Medicine, Sapporo, Japan; 3 Department of Otolaryngology, Sapporo Medical University School of Medicine, Sapporo Medical University School of Medicine, Sapporo, Japan

**Keywords:** Idiopathic pulmonary fibrosis (IPF), T follicular helper cell (Tfh cell), regulatory B cell (Breg cell), CXCR5 (C-X-C motif chemokine receptor 5), ICOS (inducible co-stimulatory molecule), PD-1 (programmed death 1), autoimmunity immunity

## Abstract

**Background:**

T follicular helper (Tfh) cells have been identified as a new category of helper T cells, which express CXCR5 on their surface and induce the production of antigen-specific antibodies. Many investigations have found morbid proliferation and/or activation of Tfh cells in systemic autoimmune and allergic diseases. It is also known that Tfh cells are regulated by regulatory B (Breg) cells in the deteriorating such diseases. Recently, CXCL13, a ligand of CXCR5, has been reported to increase in the peripheral blood and lungs of patients with idiopathic pulmonary fibrosis (IPF). This study aimed to investigate the involvement of Tfh cells and Breg cells in IPF.

**Methods:**

Peripheral blood samples were obtained from 18 patients with IPF. We isolated heparinized peripheral blood mononuclear cells and investigated the proportions of Breg cells, Tfh cells, PD-1^+^ICOS^+^ Tfh cells (activated form of Tfh cells), and the Tfh-cell subsets by flow cytometry. These cell profiles were compared with those of 21 healthy controls. Furthermore, we investigated the correlations between profiles of lymphocytes and lung physiology.

**Results:**

The median proportions of Tfh cells per total CD4^+^ T cells and of PD-1^+^ICOS^+^ proportion of Tfh cells per total Tfh cells was significantly more in the IPF patients (20.4 and 5.2%, respectively) compared with healthy controls (15.4 and 2.1%, respectively; *p* = 0.042 and *p* = 0.004, respectively). The proportion of Tfh2 cells per total Tfh cells was significantly higher and the proportion of Tfh17 was smaller in the IPF patients than healthy controls. The percentage of Breg cells to total B cells was significantly decreased in the IPF patients (median, 8.5%) compared with that in the controls (median, 19.7%; *p* < 0.001). The proportion of Breg cells was positively correlated with the annual relative change in diffusing capacity of the lungs for carbon monoxide in the IPF patients (*r* = 0.583, *p* = 0.018).

**Conclusion:**

Proliferation and activation of Tfh cells and a decrease in Breg cells were observed in the peripheral blood of patients with IPF. The profile of the Tfh-cell subset also changed. Specific humoral immunity aberration would likely underlie complicated pathophysiology of IPF.

## Background

Idiopathic pulmonary fibrosis (IPF) is a progressive and irreversible disease with a median survival time of about 3–5 years after diagnosis [1]. The etiology of IPF is still enigmatic; however, autoimmunity is considered to cause the pathogenesis of IPF [2, 3]. This is suggested by the evidence that the serum and bronchoalveolar lavage fluid of IPF patients preferentially contain antigen–antibody complexes and most patients also carry autoantibodies [4, 5]. Further, a marked correlation between specific antibodies against autoantigens (e.g., heat shock protein 70) and symptoms and prognosis of IPF patients has been demonstrated [6–9]. Recently, it was also found that IPF patients show an abnormal expression of C-X-C motif chemokine ligand 13 (CXCL13), which is a critical chemokine for the homing of B cells and T follicular helper cells (Tfh cells) to lymphoid tissues as well as inflammatory foci [10–12]. There is a strong association between circulating concentrations of CXCL13 and both clinical manifestations and disease progression of IPF [13], whereas immune settings of B cells and Tfh cells in the pathogenesis of IPF are still ill-defined.

Because B cells have an important role in specific host defense, their functional alterations cause the onset and exacerbation of autoimmune and allergic diseases. Recent studies have suggested that regulatory B (Breg) cells producing interleukin (IL)-10 and transforming growth factor-beta of a negative regulatory cytokine operate the production of antibodies in health and diseases [14, 15]. Indeed, the absence or loss of Breg cells exacerbates disease symptoms in both allergic and autoimmune diseases [15–17]. CD4^+^ T cells present in B-cell follicles, known as Tfh cells, have been established as a helper T (Th)-cell subset specialized in providing help to B cells in germinal centers (GCs). Tfh cells express the C-X-C motif chemokine receptor 5 (CXCR5) that is responsible for their migration into B-cell follicles in response to a CXCR5-specific ligand, CXCL13. Additionally, Tfh cells express co-stimulatory molecules, inducible co-stimulatory molecule (ICOS), immune-regulatory molecules, programmed death 1 (PD-1), and B-cell lymphoma-6 (BCL6) as their transcription factors. Tfh cells secrete IL-4, IL-10, and IL-21; these cytokines promote growth, differentiation, and class switching of B cells [18–20]; additionally, they are the most powerful Th cells in inducing antigen-specific antibody responses. Excessive reaction of Tfh cells causes autoantibody production, leading to autoimmune disease [21–23]. It is difficult to repeatedly examine lymphoid tissue in clinical practice; therefore, circulating CXCR5+ Th cells have been well investigated. CXCR5^+^ Th cells in the peripheral blood are reported to be associated with Tfh cells present in lymphoid tissue [19, 23, 24] and are recognized as memory Tfh cells [20]. Recent findings have clarified the relationship between Breg and Tfh cells, suggesting that Breg cells suppress Tfh-cell maturation and regulate antibody production [25].

On the basis of previous studies demonstrating that the pathogenesis of IPF is involved in autoimmunity, this study aimed to test the hypothesis that Breg and Tfh cells were associated with the pathogenesis of IPF.

## Methods

### Study population

Peripheral blood samples (20 mL) were obtained from 18 patients with IPF who attended the Sapporo Medical University Hospital from February 1st to September 30th in 2016. The diagnosis of IPF conformed to the ATS/ERS/JRS/ALAT statement in 2011 [1]. Patients who were treated with corticosteroids and/or immunosuppressive agents were excluded from this study. We used the data of 21 control subjects without previous histories of any respiratory diseases, collagen diseases, rheumatoid arthritis, and allergic diseases to compare with those of IPF patients. We obtained written informed consent from all the participants. This study was conducted in accordance with the Declaration of Helsinki and was approved by the Institutional Review Board of the Sapporo Medical University School of Medicine (approval number: 272–94).

### Antibodies

Antibodies used in the flow cytometry were anti-CD3-APC (UCHT1), anti-CD4-APC-Cy7 (RPA-T4), anti-CXCR5-PerCP-Cy5.5 (RF8B2), anti-PD-1-PE (EH12.1), and anti-ICOS-BV421 (DX29) in measuring Tfh cells and PD-1^+^ICOS^+^ Tfh cells; anti-CD3-FITC (SK7), anti-CD4-APC-Cy7 (RPA-T4), anti-CXCR5-PerCP-Cy5.5 (RF8B2), anti-CXCR3-PE-Cy7 (1C6/CXCR3), anti-CCR6-APC (11A9), anti-PD-1-PE (EH12.1), and anti-ICOS-BV421 (DX29) in investigating the Tfh-cell subset; anti-CD19-APC-Cy7 (SJ25C1), anti-CD20-PE (2H7), anti-CD24-PerCP-Cy5.5 (ML5), anti-CD27-FITC (M-T271), and anti-CD38-BV421 (HIT2) in measuring Breg cells, gated by cells negative to anti-CD3-APC (UCHT1). All antibodies were purchased from BD Biosciences.

### Lymphocyte populations and flow cytometry

We defined CD3^+^CD4^+^CXCR5^+^ lymphocytes as Tfh cells, including Tfh1 cells (CXCR3^+^CCR6^−^), Tfh2 cells (CXCR3^−^CCR6^−^) and Tfh17 cells (CXCR3^−^CCR6^+^), and Breg cells (CD3^−^CD19^+^CD24^hi^CD27^+^) [18, 26, 27]. PD-1^+^ICOS^+^ Tfh cells were regarded as an activated phenotype of Tfh cells [26]. We isolated heparinized peripheral blood mononuclear cells from fresh blood specimens by centrifugation over a discontinuous density gradient (Lympholyte-H; Cedarlane Laboratories Ltd., Canada) and investigated the proportion of Breg cells (to CD3^−^CD19^+^ B cells), Tfh cells (to CD3^+^CD4^+^ T cells), PD-1^+^ICOS^+^ Tfh cells (to all Tfh cells), and the Tfh-cell subset by flow cytometry (BD FACSCanto™ II; BD Biosciences, USA). These cell profiles among the IPF patients and healthy controls were compared by performing the Wilcoxon test.

### Correlation between the profile of lymphocytes and lung physiology

We examined the results of the pulmonary function test (PFT) performed within 3 months before or after the day of blood sampling in the IPF patients and investigated the correlation between the proportions of Tfh cells, PD-1^+^ICOS^+^ Tfh cells, Tfh-cell subsets, Breg cells, and the % predicted forced vital capacity (%FVC), % predicted diffusing capacity of the lungs for carbon monoxide (%DLCO) by using the Spearman correlation method. Regarding patients who had undergone previous PFT, we converted the change in the FVC and DLCO into annual relative change (ΔFVC and ΔDLCO) and investigated the correlation between them and the profiles of lymphocytes. When we defined FVC at the time of the cell investigation as FVC2 and FVC in the previous study as FVC1, the duration between the time of cell investigation and the previous PFT time given as X (months), the ΔFVC (%) was calculated by (FVC2 − FVC1) / FVC2 × 100 × 12 / X. Similarly, when we defined DLCO (mL/min/mmHg) at the time of the cell investigation as DLCO2 and DLCO in the previous study as DLCO1, the duration between the time of cell investigation and the previous PFT time given as X (months), the ΔDLCO (%) was calculated by (DLCO2 − DLCO1) / DLCO2 × 100 × 12 / X.

### Statistics

We used SPSS Statistics 21 (IBM Inc.) for statistical analysis. In each test, *p* < 0.05 was regarded as indicative of statistically significant differences. Data were shown as mean ± standard deviation (SD), otherwise not specifically stated.

## Results

### Subject characteristics

The subject characteristics in this study are summarized in Table 1. We compared the IPF group (14 men and 4 women, mean age; 68.2 ± 7.13 years [range, 50–79 years]) with the healthy group (7 men and 14 women, mean age; 67.8 ± 9.09 years [range, 57–88 years]). The duration from the diagnosis of IPF to the time of blood sampling varied (range, 0–35 months), and the median duration was 17.4 months. The median %FVC and %DLCO of IPF patients were 91.7% (interquartile range [IQR], 80.1–101.7%) and 56.7% (IQR, 50.6–66.0%), respectively. Sixteen patients with IPF had undergone previous PFT. The median duration between the PFT at the time of the cell investigation and the previous PFT was 24.5 months (range, 9.0–39.0 months). The median ΔFVC was − 4.25% (IQR, − 5.33−− 1.78%) and the median ΔDLCO was − 5.05% (IQR, − 12.88−− 2.70%).

**Table 1:** Subject characteristics

	Patients with IPF (*n* = 18)	Controls (*n* = 21)
Age (years) (mean ± SD [range])	68.2 ± 7.1 (50–79)	67.8 ± 9.1 (57–88)
Male/female	14/4	7/14
%FVC (median [IQR])	91.7 (80.1–101.7)	NR
%DLCO (median [IQR])	56.7 (50.6–66.0)	NR
Current or ex-smoker (%)	83.3	47.6
GAP stage (number)	I (10), II (6), III (2)	NR
6-min walk test distance (m) (median [IQR])	420 (380–500) (*n* = 11)	NR
Desaturation during 6-min walk test (number)	4 (*n* = 11)	NR

*IPF* idiopathic pulmonary fibrosis, *SD* standard deviation, %FVC forced vital capacity % predicted, *IQR* interquartile range, *NR* data not reported, %*DLCO* diffusing capacity of the lung for carbon monoxide % predicted

### Tfh cells and Tfh-cell subsets

**Fig. 1:**
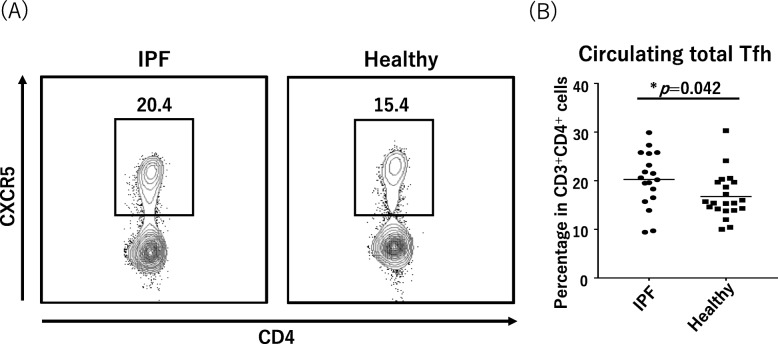
Ratios of circulating total Tfh cells in IPF and healthy cases. **a** Representative fluorescence-activated cell sorting profiles indicating total Tfh cells (CD3^+^CD4^+^CXCR5^+^). Plots were pregated on CD3^+^CD4^+^ cells and examined by the levels of CXCR5. The numbers indicate the proportion of cells in the gate. **b** The proportion of total Tfh cells in CD3^+^CD4^+^ cells is shown in the panel. Tfh, follicular helper T; IPF, idiopathic pulmonary fibrosis

**Fig. 2:**
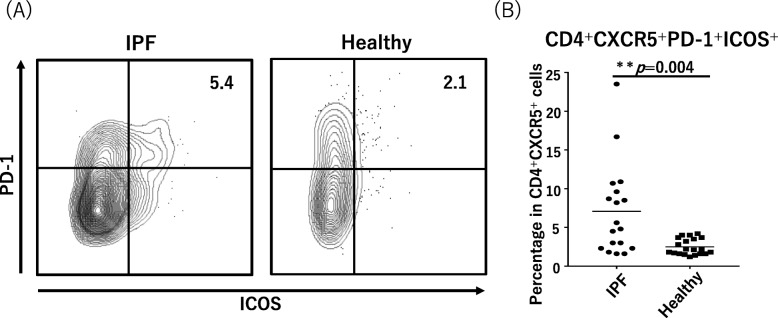
Ratios of circulating PD1^+^ICOS^+^Tfh cells in IPF and healthy cases. **a** Representative fluorescence-activated cell sorting profiles indicating PD-1^+^ICOS^+^Tfh cells. Plots were pregated on CD3^+^CD4^+^CXCR5^+^ cells and examined by the levels of PD-1 and ICOS. The numbers indicate the proportion of cells in the gate. **b** The proportion of PD-1^+^ICOS^+^Tfh cells in Tfh cells is shown in the panel. Tfh, follicular helper T; IPF, idiopathic pulmonary fibrosis

The median proportion of Tfh cells (CD3^+^CD4^+^CXCR5^+^) to total T cells (CD3^+^CD4^+^) was 20.4% (IQR, 13.4–27.2%) in the IPF patients and 15.4% (IQR, 14.2–19.7%) in the healthy controls and significantly higher in the IPF patients (*p* = 0.042; Fig. 1). Tfh cells that express the co-stimulated molecules PD-1 and ICOS are considered to be the activated forms of Tfh cells. The median proportion of PD-1^+^ICOS^+^ Tfh cells to total Tfh cells in the IPF patients (5.2% [IQR, 2.5–9.4%]) was also significantly higher than that of the healthy controls (2.1% [IQR, 1.6–3.6%], *p* = 0.004; Fig. 2).

**Fig. 3:**
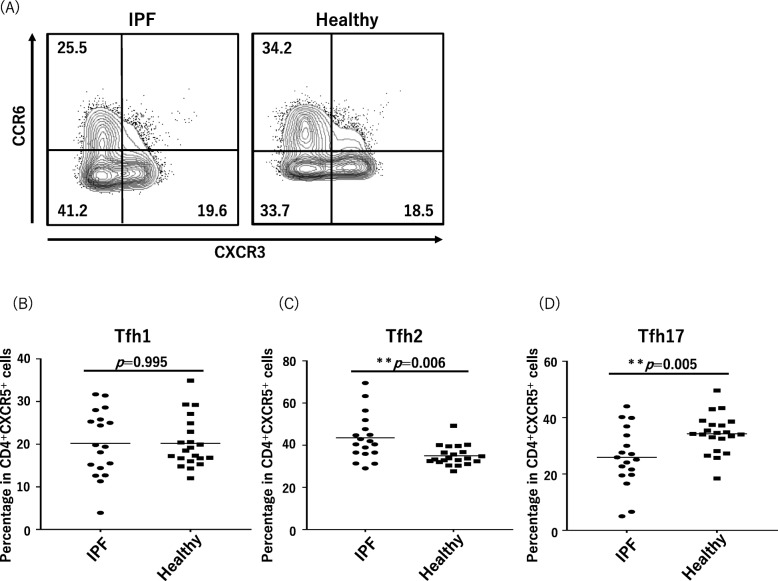
Polarization of circulating Tfh-cell subsets in IPF and healthy cases. **a** Representative fluorescence-activated cell sorting profiles indicating Tfh1 cells (CXCR3^+^CCR6^−^), Tfh2 cells (CXCR3^−^CCR6^−^), and Tfh17 cells (CXCR3^−^CCR6^+^). Plots were pregated on CD3^+^CD4^+^CXCR5^+^ cells and examined by the levels of CXCR3 and CCR6. The numbers indicate the proportion of cells in the gate. **b**–**d** The proportions of Tfh-cell subsets among all Tfh cells are shown in the panel. **b** Tfh1 cells, (**c**) Tfh2 cells and (**d**) Tfh17 cells. Tfh, follicular helper T; IPF, idiopathic pulmonary fibrosis

According to the expression profiles of two chemokine receptors, CXCR3 and CCR6, circulating Tfh cells in blood are classified into three Tfh-cell subsets including Tfh1 cells, which produces interferon-gamma like Th1 cells; Tfh2, which produces IL-4, IL-5, and IL-13 like Th2 cells; and Tfh17 cells, which produces IL-17 and IL-22 like Th17 cells. Figure 3 shows the proportions of Tfh1, Tfh2, and Tfh17 cells to total Tfh cells in the IPF patients and healthy controls. Figure 3a shows representative profiles of the flow cytometry of the IPF patients and healthy controls. The median percentage of Tfh2 cells in the IPF patients was 41.2% (IQR, 36.5–47.0%), significantly higher than that in the controls (median, 33.7% [IQR, 32.4–36.7%]; Fig. 3c). On the other hand, the proportion of the Tfh17 subset in the IPF patients (median, 25.5% [IQR, 20.2–32.8%]) was smaller than that in the healthy subjects (34.2% [IQR, 32.5–37.4%]; Fig. 3d). The percentage of Tfh1 cells was comparable between the two groups (Fig. 3b).

### Regulatory B cells

**Fig. 4:**
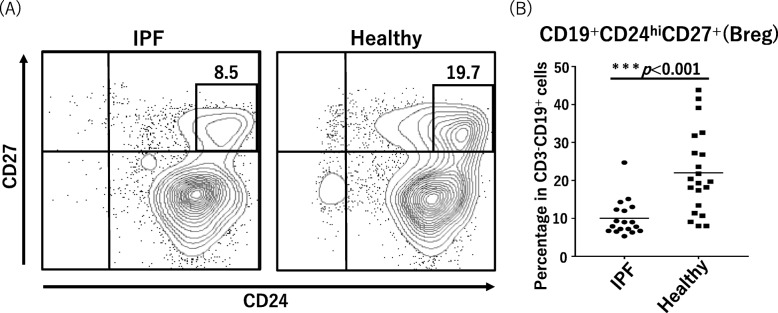
Proportions of circulating Breg cells in IPF and healthy cases. **a** Representative fluorescence-activated cell sorting profiles indicating Breg cells (CD24^hi^CD27^+^). Plots were pregated on CD3^−^CD19^+^ cells and examined according to the levels of CD24 and CD27. The numbers indicate the proportion of cells in the gate. **b** The proportion of Breg cells among all CD3^−^CD19^+^ B cells is shown in the panel. Breg, regulatory B; IPF, idiopathic pulmonary fibrosis

The proportion of Breg cells to total B cells (CD3^−^CD19^+^) was significantly decreased in the IPF patients (median, 8.5% [IQR, 6.8–12.2%]) relative to that in the controls (median, 19.7% [IQR, 13.4–27.2%], *p* = 0.001; Fig. 4).

### Correlation between the peripheral blood mononuclear cells profile and lung function

**Fig. 5:**
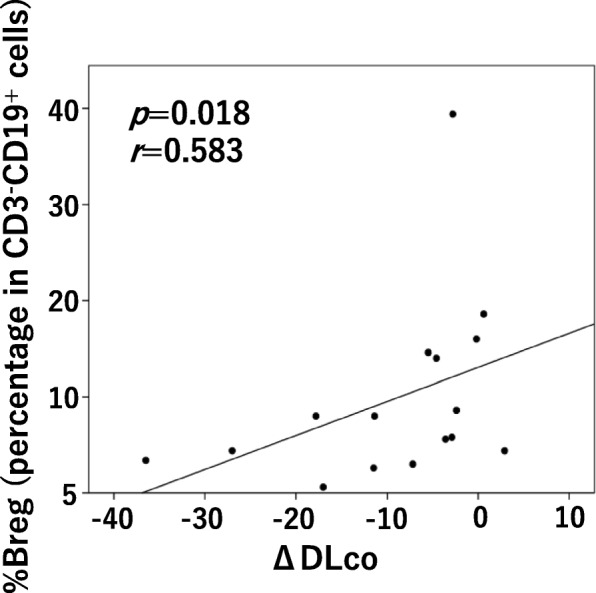
Correlation between the proportion of Breg cells and ΔDLCO in patients with idiopathic pulmonary fibrosis. ΔDLCO: means annual changes in DLCO. Breg: regulatory B; DLCO: diffusing capacity of the lung for carbon monoxide

Tfh-cell profiles (proportion of Tfh and activated Tfh, Tfh-cell subset) did not correlate with lung function and the change in lung function. Although the proportion of Breg cells to CD3^−^CD19^+^ cells correlated with neither %FVC nor %DLCO at the time of blood sampling, a positive correlation between the proportion of Breg cells and ΔDLCO was observed (*r* = 0.583, *p* = 0.018; Fig. 5).

## Discussion

In this study, we first show a possible involvement of Tfh cells and Breg cells in the pathogenesis of IPF, which is the most common type of idiopathic interstitial pneumonias. Tfh cells share CXCR5 of a chemokine receptor with B cells and have a strong capacity to evoke antigen-specific antibody responses by producing IL-21 in large quantities for the B-cell proliferation and differentiation [28, 29]. The differentiation and class switching of B cells are also promoted by the interaction of CD40 and CD40 ligand, which is presented on Tfh cells. ICOS and PD-1 as immunoregulatory molecules are highly expressed on Tfh cells and have an essential role in the differentiation and activation of Tfh cells to form GCs of lymphoid follicles. Based on the expression profile of ICOS and PD-1, circulating Tfh cells are identified as an active form (PD-1^+^ICOS^+^) and an inactive form (PD-1^−^ICOS^−^ or PD-1^+^ICOS^−^) [26]. In healthy individuals, most circulating Tfh cells rarely present ICOS and are, thus, postulated as an inactive form. In contrast, circulating ICOS^+^ Tfh cells are markedly increased in patients with autoimmune diseases, including systemic lupus erythematosus, Sjogren’s syndrome, rheumatoid arthritis, and dermatomyositis [19, 30–34]. Moreover, it is noteworthy that the proportion of ICOS^+^ Tfh cells well correlates with disease activity and autoantibody titer [19, 30–34]. Here the proportions of Tfh cells and activated Tfh cells (PD-1^+^ICOS^+^ Tfh cells) were significantly elevated in patients with IPF, suggesting that ICOS^+^ Tfh cells underlie pathologic autoimmunity responses of IPF.

Duncan SR et al. have reported that the proportion of CXCL13 and B-lymphocyte stimulating factor (BLyS) are significantly increased in lungs and peripheral blood of patients with IPF and that CXCL13 acts as a prognostic biomarker of IPF [3, 13]. There is a significant correlation between the plasma level of CXCL13 and GC activity of lymph nodes in human and mouse because CXCL13 has a cardinal role to engage Tfh cells and B cells expressing CXCR5 of a CXCL13 receptor [35]. Therefore, immune settings producing specific antibodies are probably activated in the patients with IPF. Breg cells suppress the maturation of Tfh cells and a pathologically low level of Breg cells is observed in various immune-related diseases like autoimmune diseases [36–38] as well as allergic diseases [22, 39]. So far, the fact that the patients with IPF showed a decreased level of Breg cells in comparison with healthy controls suggests that a tolerance mechanism mediated by Breg cells is not fully achieved in the regulation of activated Tfh cells in the patients with IPF [25].

Alteration of Tfh-cell subsets is observed in various immune-related diseases. Both Tfh2 cells and Tfh17 cells are predominant in autoimmune diseases, such as systemic lupus erythematosus, Sjogren’s syndrome, and systemic scleroderma [26]. The current study showed predominance of Tfh2 cells and inferiority of Tfh17 cells in the patients with IPF. IL-4 and IL-13, which Tfh2 cells are known to produce, strongly promote fibrosis [40], implying a possible involvement of Tfh2 cells in the pathogenesis of IPF. The proportion of Tfh2 cells increases and Tfh1 cells decreases in bronchial asthma and allergic rhinitis [22]. A different distribution in the Tfh-cell subsets suggests that autoimmunity is involved in the pathogenesis of IPF with mechanisms different from those of autoimmune and allergic diseases.

We examined the relationship between the results of PFT and Tfh/Breg-cell profiling measured at the same time. Results indicated that there was a positive correlation was found between the proportion of Breg cells and the annual relative change in DLCO (ΔDLCO); in other words, patients with fewer Breg cells could see worsening of DLCO. Although annual declines of FVC > 10% and/or DLCO > 15% are regarded as significant changes in the clinical course of IPF in many studies [41–43], most patients in this study showed declines below this threshold, which indicates that the clinical significance of the change in the proportion of Breg cells is unknown. Further studies using another IPF cohort with more severe diseases are necessary to verify this clinical significance. Moreover, we investigated the change in pulmonary function going back from the time of blood sampling, we need to conduct a prospective study to confirm whether or not the reduction in Breg cells will be a predictor of the deterioration of DLCO.

The clinical features of IPF patients greatly vary, and IPF might include multiple subtypes, some of which have auto-immune features. In this study, we found that large numbers of patients with IPF displayed increases of activated Tfh cells and/or decreases of Breg cells in the peripheral blood. Among these patients, a morbid immune system might play an important role in disease progression. Measuring the numbers of activated Tfh cells and Breg cells in the peripheral blood may provide a useful marker for identifying this subgroup. Anti-inflammatory therapies, such as corticosteroids, have been reported to be ineffective for the treatment of IPF [44]; however, some pulmonary diseases involving autoantibodies, such as granulomatosis with polyangitis and Goodpasture’s syndrome, are also known to be corticosteroid resistant [45]. For such diseases, treatment that suppresses the activation of B cells, such as rituximab, a human anti-CD20 monoclonal antibody, is sometime effective [46]. Furthermore, there are reports that treatment to remove autoantibodies is effective for IPF [47]; therefore a new therapeutic approach targeted to Breg cells and Tfh cells is expected.

There were several limitations in this research. The number of cases was small; there were only 18 cases of IPF and 21 cases of healthy subjects. Age was comparable among the patients with IPF and the controls; however, there was a higher proportion of females in the controls. The effects of gender on the proportions of Tfh and Breg cells and lymphocyte activation are not known, and we found no significant differences in these factors between males and females in the controls of this study (data not shown). Some issues related to the involvement of Tfh cells and Breg cells in the pathophysiology of IPF remain to be elucidated. First, where is the field of activity of these cells? Unlike interstitial pneumonia associated with autoimmune disease, morbid proliferation of lymph follicles and lymphocytic infiltration are generally negative pathological findings of IPF [48]. Since these cells might proliferate and be activated not in fibrotic lungs of IPF but in regional lymph nodes, further studies to investigate lung tissue and lymph nodes of patients with IPF will be required. As the next step, we plan to investigate the effects of morbid changes in the Tfh and Breg cells on pulmonary fibrosis by implanting activated Tfh cells (or Breg cells) into a lung fibrosis mice model. Moreover, although antigens are essential for incorporating autoimmunity, antigens in IPF are unknown. However, various types of autoantibodies have been reported to be involved in the pathogenesis of IPF, and the antigens might vary from patient to patient.

## Conclusions

In this study, we investigated the involvement of Tfh cells and Breg cells in IPF, which is fatal and has limited treatment options. The involvement of autoimmunity in fibrosis of the IPF lung is thought to be complicated. The diversity and complexity of the morbid immune settings seems to explain why corticosteroids and/or immunosuppressants are not so effective for IPF. According to this study, we can expect that drugs targeting Tfh cells and Breg cells will be developed as a new therapeutic strategy against IPF.

## Data Availability

All datasets are available from the corresponding author on reasonable request. Blood samples analyzed during the current study are not preservable.

## Abbreviations

%DLCO: diffusing capacity of the lungs for carbon monoxide % predicted
%FVC: forced vital capacity % predicted
DLCO: diffusing capacity of the lungs for carbon monoxide
FVC: forced vital capacity
ICOS: inducible co-stimulatory
IPF: idiopathic pulmonary fibrosis
IQR: interquartile range
PFT: pulmonary function test
SD: standard deviation
